# Occipital pressure sores in two neonates

**DOI:** 10.1186/s41038-015-0021-9

**Published:** 2015-11-30

**Authors:** Yi Liu, Bin Xiao, Cheng Zhang, Zhihong Su

**Affiliations:** Center of Burns and Plastic Surgery of Chinese People’s Liberation Army, Lanzhou General Hospital of Lanzhou Command, 333 Binghe South Street, Lanzhou, 730050 China

**Keywords:** Head shape, Local skin flap, Neonates, Occiput, Pressure sore

## Abstract

The preference for a specific head shape can be influenced by people’s culture, religious beliefs and race**.** Modern Chinese people prefer a “talented” head shape, which is rounded and has a long profile. To obtain their preferred head shape, some parents try to change their neonates’ sleeping position. Due to these forced sleeping positions, positional skull deformities, such as plagiocephaly, may be present during the first few months of life. In this article, we report two neonatal cases, of Hui nationality and Dongxiang nationality, with occipital pressure sores that were caused by using hard objects as pillows with the intention of obtaining a flattened occiput. The pressure sores were deep to the occipital bone and needed surgical management. These pressure sores caused wounds that were repaired by local skin flaps, after debridement, and the use of external constraints from a dense sponge-made head frame for approximately two weeks. One case recovered with primary healing after surgical operation. The other case suffered from a disruption of the sutured wound, and a secondary operation was performed to cover the wound. These occipital pressure sores are avoidable by providing guidance to the parents in ethnic minorities’ area regarding the prevention, diagnosis and management of positional skull deformity.

## Background

Occipital pressure sores are common in pediatrics, with the worldwide incidence accounting for 44.9 % of all pressure sores making it the most commonly observed pressure sore of all parts of the body [1]. Occipital pressure sores in pediatrics often occur in neonates with congenital heart disease, nervous system disease, accidental injury or an infectious disease in which they received hibernation treatment or head movement restraint. Neonates less than one-year-old are especially vulnerable, and their incidence of pressure sores accounts for 40 % of all occipital pressure sores. The majority of these pressure sores is stage I, according to the classification of pressure sore from National Ulcer Advisory Panel (NPUAP) of USA, and can be cured by change of dressings [1, 2]. In the present paper, the two cases of neonates with occipital pressure sores are different from those typically reported in pediatrics. These two cases of occipital pressures sores were caused by using bricks or tiles as pillows with the intention of changing the neonates’ head shape. These types of pressure sores were very rare, and the wounds were extremely deep. In both cases, the occipital bone was exposed and the lesions were in stage IV, which needed to be repaired by skin flap.

## Case presentation

**Fig. 1:**
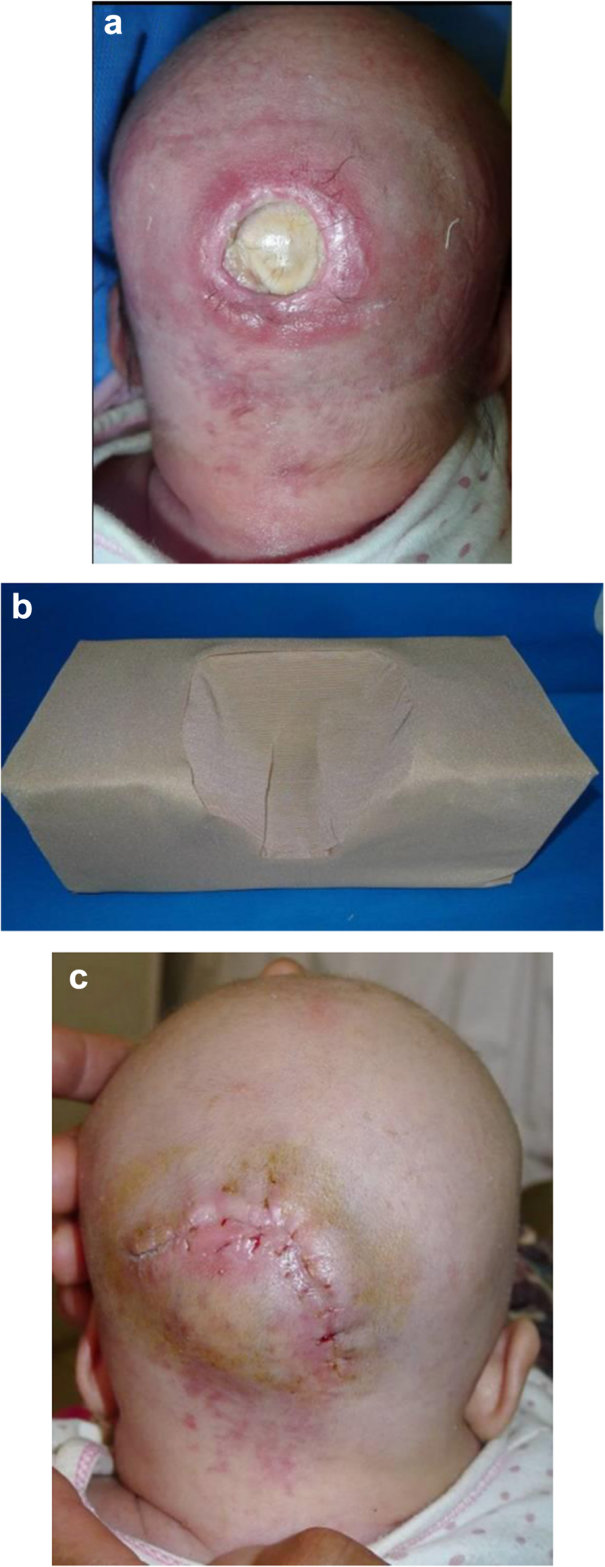
Female neonate, 20 days, pressure sore on the occiput. **a** Pre-operation. **b** Pillow made by compact sponge. **c** Repair wound with local flap, the wound healing on the first stage

Case 1 (female, 20 days old, Hui nationality) was admitted to our hospital on the 21st of March 2012 due to a skin ulcer on the occiput for the previous 10 days. After birth, the parents placed a brick wrapped in a towel under the neonate’s occiput because they thought her head shape was too rounded and unattractive. Ten days prior, the parents found a coin-sized skin ulcer on the occipital site and then went to the local hospital. No particular treatment was given because the neonate was too young. The skin lesion gradually formed a black, dry scab, which fell off two days prior to their admittance of our hospital, exposing the occipital bone. The neonate came from a secondary pregnancy and was a full-term, normal delivery. The examination on admission included the following: Temperature, 36 °C; Respiration rate, 21 times/min; Pulse, 102 beats/min; Weight, 5 kg. The baby showed normal development, modest nutritional status, normal head size, and no abnormalities upon heart and lung examination. A 2.0 cm × 2.0 cm skin lesion was located in the middle of the occipital site and deep to the occipital bone. Although the occipital periosteum disappeared, the color of the occipital bone was normal and a small quantity of purulent secretion appeared on the wound. The color of the skin surrounding the wound was normal with no swelling (Fig. 1a). Under the condition of necessary preoperative preparation, local rotational flap repairing was undertaken after debridement under general anesthesia on the 28th of March 2012. Routine anti-infection treatment was given, and a dense sponge-made head frame was used to restrain the head in a suspension position to avoid pressure on the occiput (Fig. 1b). Seven days after the surgery, the stitches were removed and the patient was discharged with primary wound healing (Fig. 1c).

**Fig. 2:**
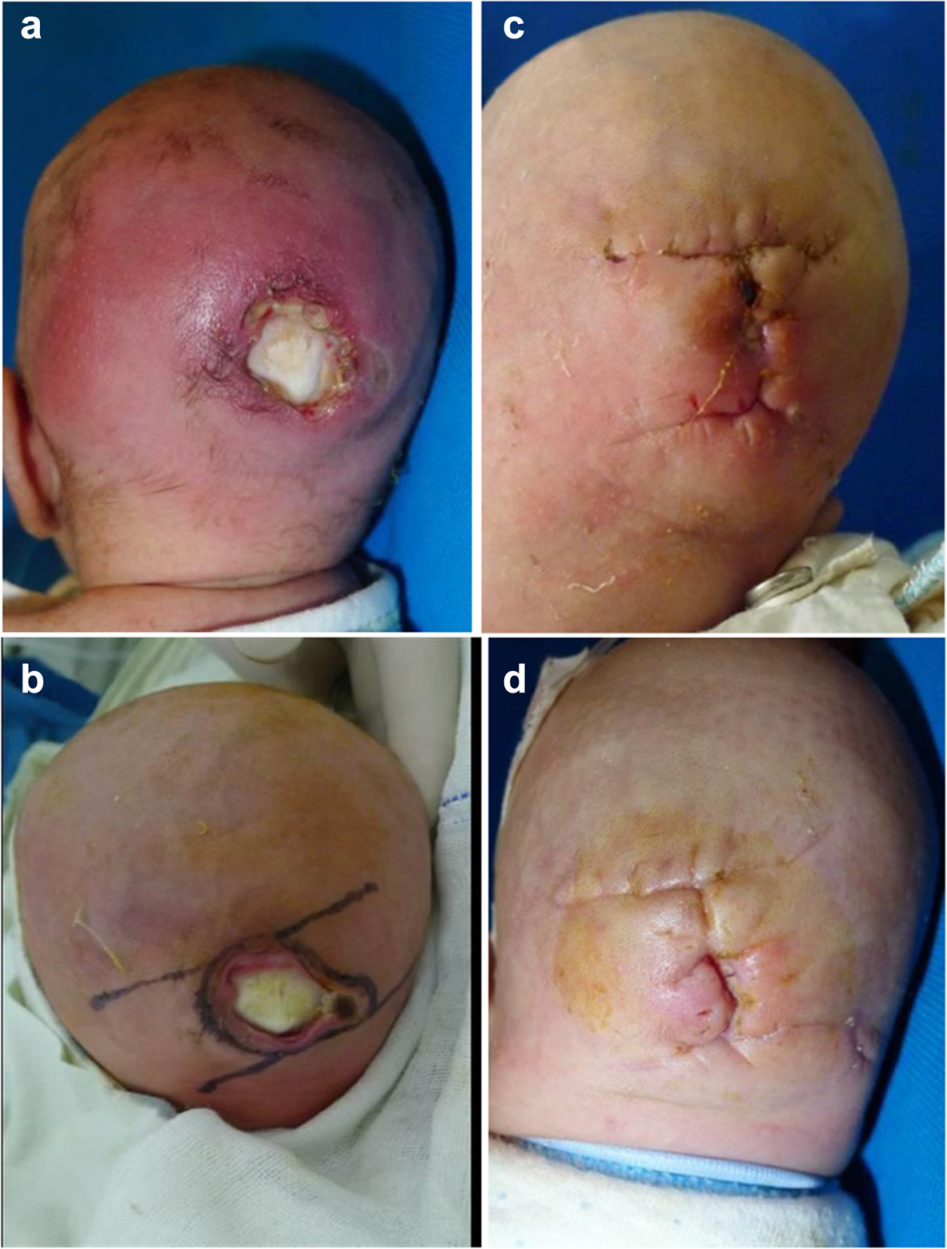
Male neonate, nine days, pressure sore on the occiput **a** Pre-operation. **b** Repair wound with local flap. **c** Wound dehiscence on the central site. **d** Wound healing after the second operation

Case 2 (male, nine days old, Dongxiang minority nationality from Linxia Dongxiang autonomous county,Gansu Province) was admitted to our hospital on the 25th of August 2013 due to the presence of a skin sore on the occipital site and exudation for five days, as well as having a fever for one day. The parents placed a tile wrapped in a towel under the neonate’s occipital site with the intention of changing his rounded occipital head shape to a flat occiput. Five days prior, the parents observed that the occipital site was pale, which was followed by the presence of a sore and exudation. Improvement was not achieved after treatment in a local clinic, and the lesion size gradually increased to exposure of the occipital bone. The neonate came from a first pregnancy with full-term normal delivery. The examination on admission included the following: Temperature, 38.5 °C; Respiration rate, 28 times/min; Pulse, 156 beats/min; Weight, 3 kg. The neonate showed normal development, modest nutritional status, normal skin color, normal head size, and no abnormalities upon heart and lung examination. There was a 2.8 cm × 1.8 cm size skin defect in the middle of the occipital site, where the occipital bone was exposed and the occipital periosteum was absent. The color of the occipital bone was normal with an irregular lesion edge. There was the presence of purulent secretion, tenderness was apparent, and the skin color around the lesion site was normal with no swelling (Fig. 2a). After systemic support, control of wound infection and preoperative preparation, occipital debridement and bilateral sliding skin flap transposition were performed to cover the exposed skull on the 30th of August 2013 (Fig. 2b). Routine hemostasis, infection control and further systemic support were given, and a dense sponge-made head frame was used to avoid occipital pressure. Lesion dehiscence was observed after stitches were removed nine days later (Fig. 2c); therefore, local suturing was undertaken on the 11th of September 2013. The stitches were removed nine days later, and the wound was healed. The patient was discharged on the 22nd of September 2013 (Fig. 2d).

## Discussion

Neonates and infants are commonly in a sleeping status wherein they lack the capability of self-protection; therefore, inappropriate infant nursing could easily lead to pressure sores with differences in nature and characteristics when compared to pressure sores in adults. The incidence rate of pressure sores in neonates and infants, from a pediatric ICU who are less than 3 months old, is 18.8 %. This incidence rate can be reduced to 6.8 % if the appropriate systemic nursing and preventive measures are implemented [3]. Inappropriate endotracheal intubation fixation also can lead to an upper lip pressure sore in low weight neonates [4]. Pressure sores are mostly seen in the upper body (i.e., occipital site) of children, especially in infants [1]. This high incidence is due to a large ratio of the head compared to the body and the occipital site as the main supporting point in supine position. Furthermore, scarce hair in infants and the relative small amount of subcutaneous tissue make the occiput sensitive to pressure and shear stress [1, 5]. Pressure sores in adults are mostly seen in patients with coma, paralysis and long-term bedding as well as among the elderly. These pressure sores are due to the inability of changing positions, with 87 % of sores occurring in the lower body; in addition, most of these patients are malnourished [6–10]. Furthermore, pressure sores caused by prolonged surgery time were also reported [11]. Liu et al., [12] classified pressure sores into three different types according to clinical characteristics: sinus type, ulcer type and mixed type. The pressure sore type in the present article is ulcer type, and the depths of the sores are deep to the skull.

Head shape refers to the outline of the head, especially a shape determined by head index. Head shape can be classified into several shapes: large, small, long, pointed and round. Head shape is mainly determined by genetic factors, and subsequent development can also have minor effects. The development of a newborn’s skull is incomplete and gaps exist among skull bones, thus allowing a certain degree of plasticity during subsequent development. Furthermore, the neck muscles of newborns are not strong enough to rotate the heavy head. Once a particular part of the head suffers from a prolonged burden of the whole head, the head type might be affected. Generally, following head development in newborns and infants, the head shape becomes stable after 3 to 4 months.

Preferences for a particular head shape have changed over time and also depend on people’s aesthetic viewpoint. In ancient times, Chinese people preferred a square shape and a large face, which projected a wealthy life and promising official career in the future. Nowadays, people prefer a “talented” head shape, which is long and rounded. Hence, Chinese parents will sometimes change their newborn’s sleeping position based on their own aesthetic preferences to obtain a certain head shape. To create a popular head shape, westerners will let their babies lie in a prone position or on their side. They especially take advantage of the prone position to prevent overgrowth of zygomatic bone, causing a higher zygomatic bone to be corrected. However, prostration can easily lead to asphyxiation and is not widely promoted unless the baby is intensively monitored during the daytime.

Although one of the studied neonates in the present report is of Hui nationality and one is of Dongxiang nationality, they are both from Chinese Muslim areas—Guanghe Hui Autonomous County, Gansu Province and Dongxiang Autonomous County, respectively—and their parents are of the Muslim faith. Both sets of parents also follow the Chinese traditional understanding of head shape that is based on the local custom of adjusting head shape through the use of hard objects as pillows, which ultimately led to the occurrence of occipital pressure sores.

The diameters of the pressure sores in the two neonates were both smaller than 2.8 cm. The lesion sizes were not large, and the surrounding scalps were normal and movable allowing a local skin flap to be used for repair after debridement. In addition, measures were taken to prevent local pressure on the occiput. The wound was cured after the first surgery in one case. In the other case, wound dehiscence occurred nine days after the removal of the stitches. From the latter result, we find that the bilateral sliding flap may not have been suitable for repair of this wound but rather a rotational flap should have been used to avoid incision dehiscence. Fortunately, the wound was healed after secondary stitches.

How does one change head shape in a medically sound manner while preventing pressure sores and related complications? Currently, exclusive baby pillow shapes can be found in infant stores. The pillow filler can be barnyard grass seed, rushes and cattail, while in the countryside, buckwheat, millet, sorghum rice or rice can be chosen as the pillow filler. Regardless of the filler, the selection principles are based on soft texture, light and durable construction, permeable to air and good moisture absorption ability. Plastic, acrylic and cotton fillers should be prohibited. One hundred percent cotton cloth is recommended for the pillowcase.

## Conclusion

Head shape change should be performed under medical supervision; otherwise, occipital pressure sores might be produced.

### Consent

Written informed consents were obtained from the patients’ parents for the publication of this report and the accompanying images.
